# Malaria vivax and Severe Thrombocytopenia in Iran

**Published:** 2010-09

**Authors:** M Metanat, B Sharifi-Mood

**Affiliations:** Research Center for Infectious Diseases and Tropical Medicine, Zahedan University of Medical Sciences, Zahedan, Iran

**Dear Editor in Chief Iranian J Parasitol**

Malaria vivax is prevalent in many regions of the world. It is more prevalent in Asia and Latin America and accounts for more than half of all malaria cases in these two regions. Clinically severe cases and complications of malaria are commonly due to *Plasmodium falciparum.* Hematological changes are nearly common in malaria infection but severe thrombocytopenia is rare in *P. vivax* (1, 2). For many years, malaria infection has been prevalent in Sistan and Baluchestan Province, southeast Iran, and we have faced with many cases of *P. vivax*. However, until 2008, we had not observed severe thrombocytopenia due to this malaria in the area. During the two last years from Aug 2007 to Sep 2009, we faced with some people along the Iran-Pakistan and Iran-Afghanistan borders, and even in the Sistan & Baluchestan Province in Iran, who had severe thrombocytopenia caused by *P. vivax*.

We evaluated all patients who were referred to our hospital in Zahedan because of vivax malaria during 2 years, from Aug 2007 to Sep 2009. Out of the 34 patients with *P. vivax* (9 females, 25 males; age range: 14- 58 years), 11 cases had a platelet count less than 50000 and among them 7 patients had a platelet count below 20000, 2 cases between 10000 to 20000 and 5 cases less than 10000. Patients who had platelet count less than 10000 had small hemorrhages under the skin and they underwent platelet transfusion.

Although, severe complications including profound thrombocytopenia are common due to falciparum malaria (3, 4) but, at now, we are facing with severe thrombocytopenia due to *vivax* malaria. It may be due to a new genotype of *P. vivax*. Non-immunological or immunological destruction of platelets are given reason for causing thrombocytopenia in such cases but the exact mechanisms involved are still not completely clear (2). Further studies are required to clarify the exact mechanism of severe thrombocytopenia especially in *P. vivax*.
